# Sociodemographic, health, and oral factors associated with health-related quality of life in children and adolescents with chronic kidney disease: a cross-sectional study

**DOI:** 10.1186/s12889-026-27958-1

**Published:** 2026-06-04

**Authors:** Nur Kodaman Dokumacıgil, Berkant Sezer, Harika Alpay, Betül Kargül

**Affiliations:** 1 Department of Pediatric Dentistry, School of Dentistry, Marmara University, İstanbul, Türkiye; 2 Department of Pediatric Dentistry, School of Dentistry, Çanakkale Onsekiz Mart University, Çanakkale, Türkiye; 3 Division of Pediatric Nephrology, Department of Pediatrics, School of Medicine, Marmara University, İstanbul, Türkiye; 4 Queen Mary University of London, London, United Kingdom

**Keywords:** Health-related quality of life, Children, Chronic kidney disease, Oral health

## Abstract

**Background:**

This study aimed to evaluate oral health parameters and health-related quality of life (HRQoL) in children and adolescents with chronic kidney disease (CKD), and compare them with non-CKD peers. Additionally, the impact of various sociodemographic, health-related, and oral health parameters on HRQoL was examined within the CKD group.

**Methods:**

In this cross-sectional observational study, 82 children with CKD were compared to 85 non-CKD controls within the same age range. Participants were evaluated through a comprehensive clinical assessment that included sociodemographic and health-related characteristics, oral hygiene habits, and oral health parameters including dental caries, oral hygiene status, gingival health, and developmental enamel defects. Both child self-report and parent proxy-report versions of the KINDL questionnaire were used to assess HRQoL. Statistical analyses were conducted to evaluate group differences and associations between variables.

**Results:**

Children with CKD had significantly lower HRQoL scores across all domains (*p* < 0.05). They also exhibited higher rates of enamel defects, gingival inflammation, and poorer oral hygiene. Although DMFT/dft scores were lower in the CKD group, ICDAS II showed a higher prevalence of early carious lesions. Multiple regression analysis revealed that HRQoL was significantly associated with CKD status, number of siblings, oral hygiene, and gingival health (*p* < 0.001).

**Conclusions:**

Oral health is strongly associated with HRQoL in children with CKD. Public health strategies should prioritize regular HRQoL monitoring and early preventive dental care to improve overall well-being in this vulnerable population.

## Introduction

Chronic kidney disease (CKD) is an often asymptomatic and difficult-to-diagnose condition that may affect over two million children and adolescents worldwide [1]. Although CKD is less common in children than in adults, it presents as a complex and burdensome condition in the pediatric population, characterized by high treatment costs and potentially devastating outcomes [2]. The rising prevalence of CKD is a growing global concern, as the disease and its management can significantly impact both patients and their families—leading to impaired growth, reduced health-related quality of life (HRQoL), and increased risks of morbidity and mortality [3, 4].

CKD is defined as the presence of kidney damage and/or a sustained reduction in glomerular filtration rate (GFR) below 60 mL/min/1.73 m² for a duration of at least three months. This condition is typically associated with proteinuria, but may also be diagnosed based on pathological findings from renal biopsy or the detection of other markers of renal damage in urine, blood, or imaging studies [5]. CKD is categorized into five stages according to the level of glomerular dysfunction [6]. As CKD progresses from Stage 1 to Stage 5 (end-stage renal disease), there is a progressive decline in GFR and an increase in renal parenchymal damage [6]. During the early stages, conservative treatments focus on controlling risk factors to prevent progression to end-stage renal disease. Stage 5 CKD, defined by a GFR < 15 mL/min/1.73 m², represents kidney failure and necessitates renal replacement therapy, such as hemodialysis, peritoneal dialysis, or kidney transplantation [7].

As kidney function deteriorates with declining GFR, children and adolescents experience an increasing burden of symptoms and treatment demands. This decline has a profound negative impact on HRQoL and is associated with a level of psychosocial and functional impairment comparable to that seen in individuals with terminal malignancies [2, 8].

CKD, depending on its progression, can adversely affect a child’s development through frequent clinical visits, polypharmacy, strict dietary restrictions, and physical changes. Moreover, it can lead to psychosocial challenges, including fatigue, anxiety, and depression [9, 10]. In addition to the direct burden of the disease, various factors such as disease-related symptoms, side effects of treatment, and the quality of family interactions can influence a child’s HRQoL [11].

Advances in the clinical management of CKD have led to improved survival rates, allowing for the more frequent observation of oral manifestations in affected patients. These manifestations are influenced by oral hygiene practices, dietary habits, environmental factors, and treatment-related variables [12]. Common oral findings in children with CKD include xerostomia, halitosis, uremic stomatitis, dysgeusia, increased dental plaque and calculus accumulation, periodontitis, tooth discoloration, developmental enamel defects, and craniofacial bone abnormalities. Additionally, drug-induced gingival hyperplasia, dysphagia, and a burning sensation in the oral cavity have also been reported [13]. Oral health issues such as periodontal disease and developmental enamel defects can negatively affect HRQoL by impairing functions like chewing and speaking, reducing self-esteem, and contributing to school absenteeism and emotional distress [2, 14]. Despite the well-documented oral manifestations of CKD, data specifically addressing the oral health status and HRQoL of Turkish children and adolescents with CKD remain scarce. In Türkiye, cross-sectional studies have reported a prevalence of low eGFR consistent with CKD stage 3 or higher at approximately 0.25% among children [1], yet the concurrent evaluation of oral health and HRQoL in this population has not been sufficiently investigated.

HRQoL is a multidimensional construct that evaluates an individual’s self-perceived well-being, encompassing physical health, psychological state, social relationships, and interactions with the surrounding environment [15]. In recent years, several pediatric-specific quality of life questionnaires have been developed to assess HRQoL using both child self-reports and parent proxy reports. Although children’s self-reports are considered the most accurate source for HRQoL evaluation, parent-reported assessments provide valuable complementary insights [16]. Various versions of HRQoL instruments—tailored to different age groups and disease contexts—have been adapted into multiple languages and validated for use in a wide range of acute and chronic pediatric conditions [3, 17].

The primary aims of this study were to evaluate the HRQoL scores of children and adolescents with CKD, including those undergoing dialysis and kidney transplantation, and to investigate the associations between HRQoL and sociodemographic characteristics as well as oral health status indicators. Additionally, the study aimed to compare HRQoL outcomes with those of a non-CKD group and to assess the level of agreement between children’s self-reported HRQoL and parent/caregiver proxy assessments. The null hypothesis of the study was that there would be no significant differences in oral health or HRQoL scores between children and adolescents with CKD and their non-CKD peers.

## Methods

A cross-sectional, observational study was conducted to determine the HRQoL and its relationship with sociodemographic and oral hygiene indicators in a group of children and adolescents diagnosed with CKD and undergoing dialysis or transplantation.

### Ethical approval

The study was undertaken after obtaining ethical clearance from the Marmara University School of Medicine Clinical Research Ethics Committee with a protocol number of 09.2022.961, according to the Helsinki Declaration. The details of the study were explained with consideration of the participants’ ages and cognitive capacities, and the informed consent form was signed by both the patient and their parents and/or caregivers.

### Participants

**Fig. 1:**
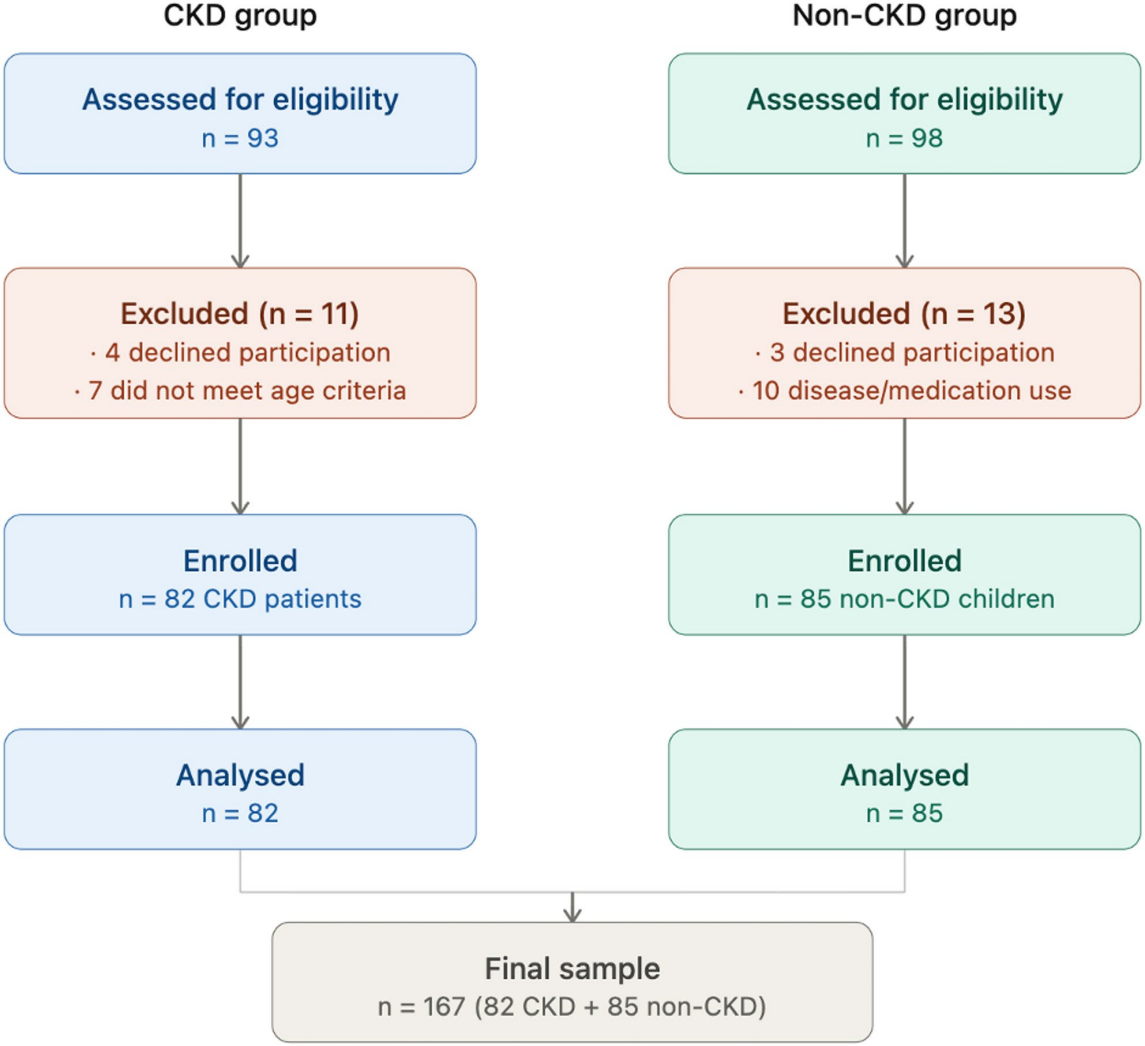
Participant recruitment flow diagram

The study was conducted between 2022 and 2023 at the Pediatric Nephrology Unit of the Department of Pediatrics, Marmara University Faculty of Medicine, and Istanbul Pendik Training and Research Hospital. Of the 93 CKD patients assessed for eligibility, 11 were excluded (4 declined participation and 7 did not meet age criteria), resulting in 82 enrolled patients. The non-CKD group comprised 85 of the 98 children assessed for eligibility; 13 were excluded (3 declined participation and 10 were ineligible due to systemic disease or regular medication use) (Fig. 1). These individuals were either siblings of children with CKD or came from similar socioeconomic and demographic backgrounds and had presented to the same unit for routine check-ups.

Inclusion criteria for the study were as follows: being between 8 and 16 years of age; having been diagnosed with CKD at least one year prior to participation (stages 1–5, as defined by the Kidney Disease: Improving Global Outcomes [KDIGO] guidelines) [18]; no history of acute systemic illness within the past 30 days; absence of cognitive impairments that could hinder comprehension or participation; no comorbid diseases or conditions; no ongoing orthodontic treatment; and no history of periodontal treatment within the previous six months [19].

### Sampling procedures and study power

The sample size calculation was performed using G*Power 3.1.9.2 (University of Düsseldorf, Germany) statistical software. The calculation was based on a two-tailed Student’s t-test for independent samples, with a significance level (α) of 5% and a statistical power of 85%. The estimated effect size (Cohen’s d) was determined as 0.455, based on the study by Silva et al. [2], which identified a significant relationship between poor oral hygiene and HRQoL. According to this calculation, a minimum of 82 participants per group (a total of 164 participants) was required to achieve 85% power. Our study includes 82 children and adolescents with chronic kidney disease (CKD) and 85 non-CKD children. These values meet the required sample size and provide sufficient statistical power to detect significant differences between the groups.

### Data collection and variables

Age, sex, number of siblings, stage of CKD, body mass index (BMI), duration of the disease, history of dialysis and transplantation, frequency of pediatric nephrology clinic visits, and the presence of any additional systemic diseases or conditions other than CKD were recorded using standardized medical history forms. This data was collected for both children and adolescents with CKD and their non-CKD counterparts by a pediatric nephrologist and a pediatric dentist. In addition, information regarding oral health behaviors—including tooth brushing frequency, flossing habits, and the number of annual dental visits—was documented prior to the intraoral examination.

### Intraoral examination

Intraoral examinations for all participants were performed by the same pediatric dentist at the Pediatric Nephrology outpatient clinic, with the child in a standing position under natural light, using a periodontal probe and a mouth mirror. The examinations were conducted within the nephrology clinic setting rather than a dental faculty environment, as the CKD patient group represents an infection-susceptible population for whom unnecessary clinical transfers were considered inadvisable. The clinical assessment included the evaluation of dental caries using the Decayed, Missing, and Filled Teeth (DMFT/dft) Index [20] and the International Caries Detection and Assessment System II (ICDAS II) [21]; developmental enamel defects using the Developmental Defects of Enamel (DDE) Index [22]; and molar incisor hypomineralization (MIH) according to established diagnostic criteria [23]. In addition, dental plaque and calculus accumulation were evaluated using the Debris Index (DI) and Calculus Index (CI) of the Simplified Oral Hygiene Index (OHI-S) [24], while gingival health was assessed using the Modified Gingival Index (MGI) [25].

Dental caries prevalence and severity were measured using the DMFT/dft Index, in which the sum of decayed, missing, and filled teeth (D + M+F / d + m+f) provides a final score. In cases where retained primary teeth were present, only the caries status of permanent teeth was recorded, in accordance with WHO recommendations [20]. Missing primary teeth were not included due to physiological exfoliation. Mean DMFT/dft values were calculated by summing the individual scores and dividing by the total number of participants [20]. The ICDAS II criteria were applied to assess the presence and severity of carious lesions at the surface level. For each tooth, the highest score recorded on any surface was used as the representative ICDAS II score. Scores were categorized as follows: “0” = sound tooth surface; “1–2” = initial lesions; “3–4” = moderate lesions; and “5–6” = severe carious lesions [21].

DDE were assessed using the DDE Index, which identifies enamel opacities and hypoplasia across the entire tooth surface through visual and intraoral inspection. A participant was classified as having DDE if at least one affected tooth exhibited a defect [22]. Teeth with severe dental caries were excluded from the DDE assessment [26]. The diagnosis of MIH was based on the presence of post-eruptive enamel breakdown, hypersensitivity, demarcated opacities, or atypical restorations, and was recorded as either “present” or “absent” according to established diagnostic criteria [23].

Oral hygiene status was evaluated using the OHI-S, which comprises the DI and CI components. The DI measures plaque accumulation, while the CI assesses calculus formation. Scores were recorded on the buccal/facial surfaces of the following index teeth: permanent maxillary right first molar, maxillary right central incisor, maxillary left central incisor, maxillary left first molar, mandibular left first molar, mandibular left central incisor, mandibular right central incisor, and mandibular right first molar. Individual DI and CI scores were calculated by summing the scores for all examined surfaces and dividing by the number of surfaces assessed. The total OHI-S score was determined by summing the DI and CI values, with higher scores indicating poorer oral hygiene. According to the classification by Greene and Vermillion [24], oral hygiene status is defined as follows: 0.0–1.2 = good, 1.3–3.0 = fair, and 3.1–6.0 = poor.

Gingival health was assessed using the MGI, which is designed to evaluate mild to moderate gingival inflammation without the use of probing to avoid tissue irritation [25]. Each tooth was visually examined, and the overall MGI score was calculated by averaging the scores across all examined teeth, providing a general measure of gingival health status.

### Health-related quality of life instrument

The KINDL scale is widely used to assess HRQoL in children with various chronic diseases and/or developmental disorders, as well as in their parents, to determine which aspects of life are most impacted by the illness and its treatment [27]. The Turkish version of the KINDL, utilized for both child self-reports and parent proxy-reports in the current study, is a validated instrument that has been previously translated and culturally adapted for use in relevant Turkish pediatric age groups [28]. The KINDL includes three age-specific versions designed for children aged 4–7, 8–12, and 13–16 years [27], and evaluates six core dimensions of HRQoL: physical well-being, emotional well-being, self-esteem, family, friends, and school functioning. In addition, a chronic disease module provides a specific assessment of HRQoL in the context of chronic health conditions [29]. The questionnaire was completed by 82 children diagnosed with CKD and 82 of their parents.

To achieve a sufficient sample size, the Kid-KINDL (for ages 8–12) and Kiddo-KINDL (for ages 13–16) versions were administered according to the participant’s age and subsequently pooled for analysis. Both versions share an identical item structure (24 core items and a six-item chronic disease module), use the same 5-point Likert response format (1 = Never, 2 = Rarely, 3 = Sometimes, 4 = Often, 5 = Always), and produce scores on the same 0–100 scale, with higher scores indicating better HRQoL [30], thereby supporting their combined use. Age was included as a covariate in the multiple linear regression model to account for any potential differences attributable to the use of different age-specific versions. Both versions of the questionnaire were administered in paper-based format. Children and adolescents completed the self-report version independently, while at least one parent or caregiver simultaneously completed the proxy-report version.

### Statistical analysis

Descriptive statistics were presented as frequency (%) for categorical variables and as the median (IQR, interquartile range) for continuous variables that were not normally distributed, or as the mean (SD, standard deviation) for normally distributed continuous variables. The assumption of normality was assessed using the Shapiro-Wilk test. Comparisons between non-CKD and CKD patients were conducted using the Student t test, Mann-Whitney U test, Chi-square test, and Linear-by-Linear association test. Patient and parent HRQoL scores were compared using the Wilcoxon signed-rank test. Total HRQoL scores were analyzed based on patients’ characteristics using the Mann-Whitney U test or the Kruskal-Wallis test, with Bonferroni-adjusted p-values applied for multiple comparisons. The relationships between HRQoL scores and other variables were examined using Spearman’s rank correlation analysis. Variables significantly associated with total HRQoL scores or CKD status were included in a multiple linear regression model. Model diagnostics, including assessments of autocorrelation, multicollinearity, normality of standardized residuals, and identification of extreme observations, were conducted. Variables with a high variance inflation factor (CI and DI) and three extreme observations were excluded from the final model. Statistical significance was set at *p* < 0.05, and all analyses were performed using the open-source statistical software JASP v.0.16.3.0 and R.

## Results

This study included 82 CKD patients and 85 non-CKD children. The mean CKD duration was 9.56 ± 3.73 years. Thirty patients (36.6%) had CKD level of 1–3 and remaining 52 (63.4%) patients had CKD level of 4 or 5. The dialysis was present in 40 (48.8%) and transplantation was present in 8 (9.8%) CKD patients.

There were no significant differences between CKD patients and non-CKD children in terms of age, sex, number of siblings, and BMI status (*p* > 0.05) (Table 1). Ninety-three children were boys (55.7%), and there were 64 children who had 3 or more siblings (38.3%). According to BMI categorization, there were 84 (50.3%) underweight, 64 (38.3%) normal, and 19 (11.4%) overweight children.

**Table 1:** Comparison of sociodemographic, oral health, and HRQoL characteristics between children with CKD and non-CKD controls

		CKD	Non-CKD	*p*-value
Age ^a^		11.79 ± 3.45	11.14 ± 2.89	0.187^c^
Sex	Male	44 (53.66%)	49 (57.65%)	0.604^d^
	Female	38 (46.34%)	36 (42.35%)	
Number of Siblings	None	4 (4.88%)	14 (16.47%)	0.095^d^
	1	16 (19.51%)	18 (21.18%)	
	2	27 (32.93%)	24 (28.24%)	
	3 or more	35 (42.68%)	29 (34.12%)	
BMI	Underweight	48 (58.54%)	36 (42.35%)	0.092^d^
	Normal	25 (30.49%)	39 (45.88%)	
	Overweight	9 (10.98%)	10 (11.76%)	
Toothbrushing	Never	31 (37.8%)	9 (10.59%)	< 0.001^d^*
	Once a day	40 (48.78%)	29 (34.12%)	
	Twice a day or more	11 (13.41%)	47 (55.29%)	
Dental floss use	Yes	3 (3.66%)	29 (34.12%)	< 0.001^d^*
Dental visit per year	None	46 (56.1%)	5 (5.88%)	< 0.001^d^*
	Once	31 (37.8%)	66 (77.65%)	
	Twice or more	5 (6.1%)	14 (16.47%)	
DMFT/dft^b^		0 (0–2)	7 (4–10)	< 0.001^d^*
ICDAS	Healthy	39 (47.56%)	8 (9.41%)	< 0.001^e^*
	Initial	20 (24.39%)	14 (16.47%)	
	Medium	12 (14.63%)	21 (24.71%)	
	Severe	11 (13.41%)	42 (49.41%)	
DDE	Absence	10 (12.2%)	53 (62.35%)	< 0.001^e^*
	Demarcated	43 (52.44%)	19 (22.35%)	
	Diffuse	11 (13.41%)	10 (11.76%)	
	Hypoplasia	13 (15.85%)	3 (3.53%)	
	Other defects	5 (6.1%)	0 (0%)	
MIH	Presence	12 (14.63%)	10 (11.76%)	0.684^e^
DI^b^		1.33 (1-1.83)	0.33 (0-0.66)	< 0.001^f^*
CI^b^		0.66 (0.16–1.33)	0 (0–0)	< 0.001^f^*
OHI-S^b^		1.92 (1-3.33)	0.33 (0-0.66)	< 0.001^f^*
MGI	Normal	8 (9.76%)	52 (61.18%)	< 0.001^e^*
	Very mild	17 (20.73%)	23 (27.06%)	
	Mild	15 (18.29%)	8 (9.41%)	
	Moderate	27 (32.93%)	2 (2.35%)	
	Severe	15 (18.29%)	0 (0%)	
HRQoL^b^	Physical well-being	3 (3-3.5)	4 (3.5–4.25)	< 0.001^f^*
	Emotional well-being	3 (3-3.5)	4 (3.75–4.25)	< 0.001^f^*
	Self-esteem	2.75 (2.5-3)	3.5 (3–4)	< 0.001^f^*
	Family	3.5 (3–4)	4 (4-4.5)	< 0.001^f^*
	Friends	3 (2.75–3.5)	4 (3.5–4.5)	< 0.001^f^*
	School	3 (2.75–3.25)	3.5 (3.25-4)	< 0.001^f^*
	Disease perception	2.88 (2.25–3.25)	–	n.a.
	Total	3.03 (2.89–3.26)	3.25 (3.07–3.57)	< 0.001^f^*

*CKD* chronic kidney disease, *BMI* body mass index, *DMFT/dft* decay missing filled index, *ICDAS* international caries detection and assessment system, *DDE* developmental defects of enamel, *DI* debris index, *CI* calculus index, *OHI-S* simplified oral hygiene index, *MGI* modified gingival index, *MIH* molar incisor hypomineralization, *HRQoL* health related quality of life

Descriptives are reported with frequency (%), ^a^mean ± SD or ^b^median (IQR). ^c^Student t test ^d^Chi-square test; ^e^Linear by Linear association; ^f^Mann-Whitney U test

*Statistically significant at *p* < 0.001 level

Toothbrushing and dental floss use were less frequent in CKD patients than the non-CKD children (*p* < 0.001). Yearly dental visit frequency and DMFT/dft [CKD: 0 (IQR = 0–2) vs. non-CKD: 7 (IQR = 4–10)] were significantly lower in CKD patients than the non-CKD children (*p* < 0.001). Those with healthy ICDAS were much more prevalent in CKD patients, while severe conditions were much more frequent in the non-CKD children (*p* < 0.001). The abnormal DDE and MGI conditions were more frequent in CKD patients while normal DDE and MGI conditions were more frequent in the non-CKD children (*p* < 0.001). The poor dental health indicators of DI, CI, and OHI-S were significantly higher in CKD patients compared to non-CKD children (*p* < 0.001). The HRQoL scores were significantly lower in CKD patients than the non-CKD children (*p* < 0.001) (Table 1).

There were no significant differences between children and their parents’ HRQoL scores of total, physical well-being, emotional well-being, family, friends and school (*p* > 0.05). Children had significantly lower self-esteem HRQoL than their parents (*p* < 0.001). Those children with CKD had lower disease perception HRQoL compared to their parents (*p* < 0.001) (Table 2).

**Table 2:** Comparison of HRQoL scores between child self-reports and parent proxy-reports in CKD patients

HRQoL scores	Children	Parent	*p*-valueᵃ
	Median (IQR)	Median (IQR)	
Physical well-being	3.5 (3–4)	3.5 (3–4)	0.393
Emotional well-being	3.5 (3–4)	3.5 (3.25–4)	0.735
Self-esteem	3 (2.75–4)	3.5 (3–4)	0.008*
Family	4 (3.25–4.25)	3.75 (3–4.5)	0.378
Friends	3.5 (3–4)	3.5 (3–4)	0.702
School	3.25 (3–4)	3.25 (2.75–4)	0.612
Disease perception	2.88 (2.25–3.25)	3 (2.60–3.50)	0.003*
Total	3.14 (2.96–3.43)	3.18 (2.96–3.54)	0.290

*HRQoL* health related quality of life, *IQR* interquartile range

Disease perception HRQoL score compared in CKD patients

ᵃWilcoxon test *statistically significant at *p* < 0.01 level

No significant differences were observed in the HRQoL total score based on sex, BMI, CKD level, transplantation and dialysis status, or ICDAS scores (*p* > 0.05) (Table 3). However, children without siblings reported significantly higher HRQoL scores compared to those with two (*p* = 0.002) or at least three siblings (*p* = 0.007). Additionally, children who never brushed their teeth had significantly lower HRQoL scores than those who brushed at least twice a day (*p* = 0.001). Those who used dental floss exhibited significantly higher HRQoL scores compared to those who did not (*p* = 0.025). Children who did not visit a dental clinic in the past year had significantly lower HRQoL scores than those who visited once (*p* = 0.020) or at least twice (*p* = 0.043). Furthermore, children without developmental dental defects (DDE) had significantly higher HRQoL scores than those with diffuse opacities (*p* = 0.011) or hypoplasia (*p* = 0.042). Those with a normal MGI condition reported significantly higher HRQoL scores compared to those with very mild (*p* = 0.035), mild (*p* = 0.027), moderate (*p* = 0.001), and severe (*p* = 0.025) MGI conditions. Finally, children with MIH showed significantly lower HRQoL scores compared to those without MIH (*p* = 0.040) (Table 3).

**Table 3:** HRQoL total scores according to sociodemographic, clinical, and oral health characteristics in CKD patients

		HRQoL Total	*p*-value
		Median (IQR)	
Sex	Male	3.14 (2.96–3.46)	0.546ᵃ
	Female	3.14 (2.96–3.36)	
Number of siblings	None	3.59 (3.07–3.79)	0.001ᵇ*
	1	3.23 (3.04–3.43)	
	2	3.07 (2.89–3.39)	
	3 or more	3.07 (2.9–3.27)	
BMI	Underweight	3.08 (2.93–3.41)	0.486ᵇ
	Normal	3.18 (3-3.43)	
	Overweight	3.21 (2.96–3.43)	
CKD level	1–3	3.05 (2.96–3.29)	0.143ᵃ
	4–5	3 (2.88–3.24)	
Transplantation	Presence	3.14 (2.93–3.3)	0.373ᵃ
	Absence	3.03 (2.89–3.21)	
Dialysis	Presence	3 (2.88–3.14)	0.066ᵃ
	Absence	3.08 (2.89–3.3)	
Toothbrushing	Never	3.04 (2.82–3.19)	0.001ᵇ*
	Once a day	3.14 (2.96–3.29)	
	Twice a day or more	3.36 (3.04–3.64)	
Dental floss use	Yes	3.38 (3.07–3.61)	0.025ᵃ*
	No	3.11 (2.95–3.32)	
Dental visit per year	None	3.04 (2.89–3.21)	0.009ᵇ*
	Once	3.21 (3-3.43)	
	Twice or more	3.31 (3-3.71)	
ICDAS	Healthy	3.07 (2.89–3.29)	0.253ᵇ
	Initial	3.19 (2.96–3.61)	
	Medium	3.25 (3.04–3.39)	
	Severe	3.14 (3-3.45)	
DDE	Normal	3.25 (3.07–3.64)	0.002ᵇ*
	Demarcated	3.07 (2.96–3.32)	
	Diffuse	2.96 (2.86–3.25)	
	Hypoplasia	2.97 (2.80–3.19)	
	Other defects	3.16 (3.04–3.31)	
MGI	Normal	3.36 (3.07–3.68)	< 0.001ᵇ*
	Very mild	3.09 (2.91–3.38)	
	Mild	3.07 (2.93–3.29)	
	Moderate	3.04 (2.89–3.16)	
	Severe	3.04 (2.9–3.14)	
MIH	Presence	3.04 (2.89–3.21)	0.040ᵃ*
	Absence	3.16 (2.96–3.46)	

*HRQoL* health related quality of life, *IQR* interquartile range, *BMI* body mass index, *CKD* chronic kidney disease, *ICDAS* international caries detection and assessment system, *DDE* developmental defects of enamel, *MGI* modified gingival index, *MIH* molar incisor hypomineralization

Descriptives are reported with median (IQR). ᵃ Mann-Whitney U test; ᵇ Kruskal-Wallis test. *Statistically significant at *p* < 0.05 level

The correlation relationship between HRQoL scores is presented in Table 4. The total HRQoL score was positively correlated with other HRQoL scores in both CKD patients and non-CKD children (*p* < 0.001). The high physical well-being of the children was associated with the high Friends HRQoL (*p* < 0.05). The emotional well-being of the children was positively correlated to family HRQoL, friends HRQoL and school HRQoL (*p* < 0.05). Self-esteem HRQoL was related with the better HRQoL in family, friends, and school (*p* < 0.05). The family and friends HRQoL scores were positively correlated (*p* < 0.001) and the friends HRQoL was significantly associated with school HRQoL (*p* < 0.001). The family HRQoL was positively correlated with CI in both CKD patients and non-CKD children (*p* < 0.05) (Table 4). The lower friend HRQoL was associated with the increased duration of CKD ($\:{r}_{s}\:$= -0.29; *p* = 0.007). The lower DMFT/dft of CKD patients was associated with the lower self-esteem HRQoL ($\:{r}_{s}\:$= 0.27; *p* = 0.014), while the high OHI-S ($\:{r}_{s}\:$= 0.24; *p* = 0.032) were correlated with higher family HRQoL (Table 4). The lower HRQoL and lower physical well-being of non-CKD children are associated with the higher DMFT/dft, DI, CI, and OHI-S (*p* < 0.05). The higher DMFT of non-CKD children was correlated with lower emotional well-being and school HRQoL (*p* < 0.01). The friends HRQoL in non-CKD children were negatively correlated with DI, CI and OHI-S (*p* < 0.01). The lower self-esteem and family HRQoL scores were associated with the higher OHI-S in non-CKD children (*p* < 0.05) (Table 4).

**Table 4:** Spearman rank correlations between HRQoL scores and clinical variables in CKD patients and non-CKD children

CKD patients	Total	PWB	EWB	SE	Family	Friends	School	DP
PWB	0.454***							
EWB	0.435***	0.176						
SE	0.613***	0.283**	0.117					
Family	0.642***	0.207	0.359***	0.330**				
Friends	0.732***	0.311**	0.504***	0.360***	0.390***			
School	0.529***	0.212	0.278*	0.481***	0.027	0.489***		
DP	0.360***	-0.019	0.084	0.167	0.074	0.143	0.075	
Age	-0.146	0.052	0.171	-0.123	-0.125	-0.102	0.023	-0.091
CKD Duration	-0.139	-0.012	0.096	-0.118	0.024	-0.294**	-0.106	-0.033
DMFT/dft	0.177	0.014	-0.041	0.269*	0.127	0.110	0.055	0.027
DI	0.083	-0.072	0.159	0.076	0.145	0.161	0.059	0.002
CI	0.111	-0.080	0.085	0.051	0.233*	0.045	0.024	0.061
OHI-S	0.141	-0.072	0.148	0.077	0.237*	0.144	0.058	0.057

Non-CKD children	Total	PWB	EWB	SE	Family	Friends	School	
PWB	0.625***							
EWB	0.693***	0.322**						
SE	0.631***	0.166	0.330**					
Family	0.723***	0.496***	0.435***	0.232*				
Friends	0.806***	0.360***	0.508***	0.515***	0.485***			
School	0.704***	0.413***	0.428***	0.426***	0.436***	0.429***		
Age	0.060	0.069	0.122	0.032	0.042	0.085	0.010	
DMFT/dft	-0.336**	-0.351***	-0.289**	-0.171	-0.143	-0.208	-0.328***	
DI	-0.321**	-0.325**	-0.096	-0.206	-0.199	-0.375***	-0.175	
CI	-0.276*	-0.244*	-0.009	-0.174	0.227*	-0.305**	-0.151	
OHI-S	-0.342***	-0.352***	-0.092	-0.230*	-0.219*	-0.395***	-0.170	

*Abbreviations*: *CKD* chronic kidney disease, *PWB* physical well-being, *EWB* emotional well-being, *SE* self-esteem, *DP* disease perception, *DMFT/dft* decay missing filled index, *DI* debris index, *CI* calculus index, *OHI-S* simplified oral hygiene index. rs: Spearman rank correlation coefficient

*p < 0.05; **p < 0.01; ***p < 0.001

**Fig. 2:**
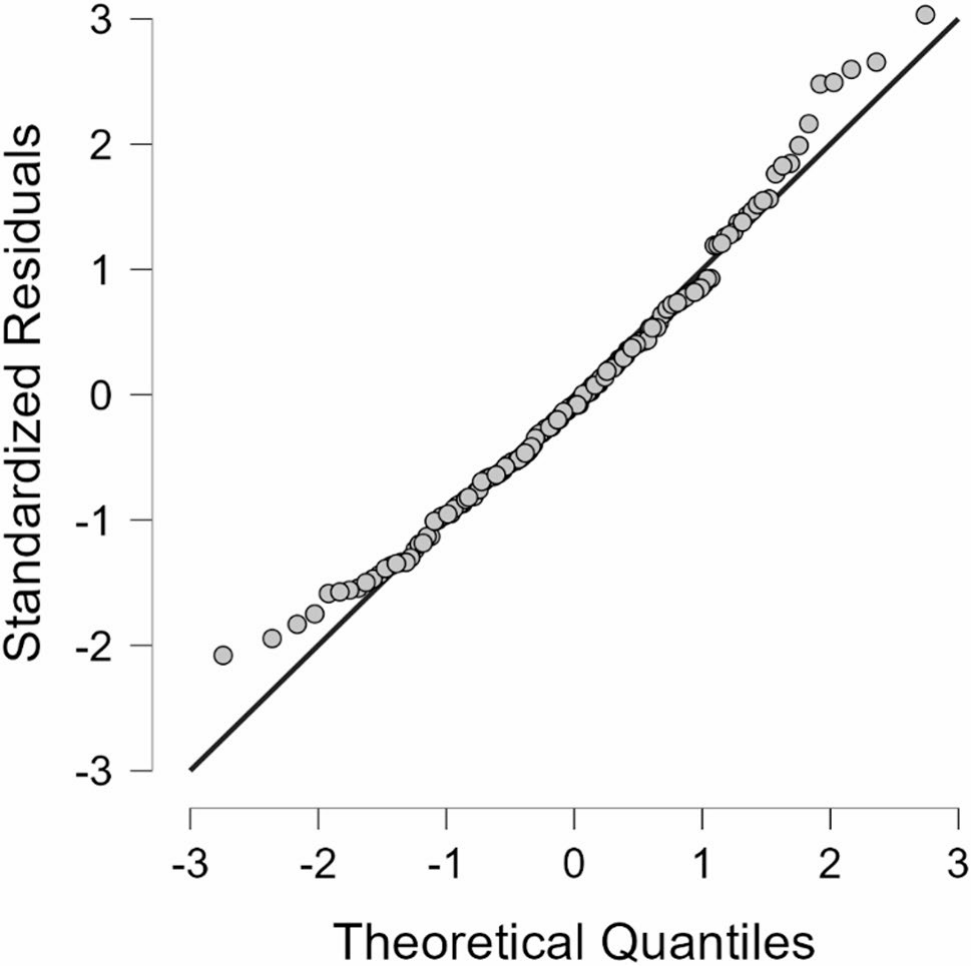
Q-Q Plot standardized residuals

The multiple regression predicting total HRQoL score is presented in Table 5. The model included the predictors of patient group, age, number of siblings, OHI-S, and MGI status. The model explained the 21.8% of the variation in the total HRQoL score of children (*R* = 0.569; R² = 0.324; Adjusted R² = 0.218; F (22, 141) = 3.067; *p* < 0.001). Total HRQoL scores were 0.244 points lower in children with CKD compared to non-CKD children (*p* = 0.030). In addition, children with two siblings had HRQoL scores that were 0.289 points lower (*p* = 0.004), while those with three or more siblings had scores that were 0.215 points lower (*p* = 0.034), compared to children without siblings. A one-unit increase in OHI-S score was associated with a 0.068-point increase in total HRQoL score (*p* = 0.029). Conversely, children with very mild MGI had HRQoL scores that were 0.197 points lower (*p* = 0.010), and those with mild MGI had scores that were 0.238 points lower (*p* = 0.016), compared to children with normal MGI status (Table 5). The model showed good fit, and Q-Q plot of the standardized residuals is presented in Fig. 2.

**Table 5:** Multiple linear regression analysis of factors associated with total HRQoL scores in CKD patients and non-CKD children

Covariate		Unstd. B	SE (B)	Std. Bᵃ	t	*p*	95% CI
Patient group^b^	CKD	-0.244	0.112		-2.187	0.030*	(-0.023–0 0.012)
Age		-0.006	0.009	-0.050	-0.645	0.520	(-0.466 – -0.023)
Number of siblings^c^	2	-0.289	0.099		-2.917	0.004*	(-0.485 – -0.093)
	3 or more	-0.215	0.101		-2.137	0.034*	(-0.414 – -0.016)
OHI-S		0.068	0.031	0.229	2.210	0.029*	(0.007–0.128)
MGI^d^	Very mild	-0.197	0.075		-2.618	0.010*	(-0.346 – -0.048)
	Mild	-0.238	0.097		-2.445	0.016*	(-0.431 – -0.046)
Constant		3.636	0.168		21.666	< 0.001	(3304–3968)

*CKD* chronic kidney disease, *unstd* unstandardized, *SE* standard error, *CI* confidence interval, *OHI-S* simplified oral hygiene index, *MGI* modified gingival index

ᵃStandardized beta coefficients can only be computed for continuous predictors. ^b^: reference non-CKD; ^c^: reference none; ^d^: reference normal

The model included *n* = 164 pediatric patients. Model yielded R = 0.569, R² = 0.324, Adjusted R² = 0.218; F(22, 141) = 3.067; *p* < 0.001

*Statistically significant at *p* < 0.05 level

## Discussion

In this study, sociodemographic characteristics, oral health status, and HRQoL were comprehensively compared between children and adolescents with CKD—including those undergoing dialysis and renal transplantation—and their non-CKD peers. The findings revealed significant disparities across these groups, underscoring the need for a multidisciplinary approach in the medical and dental care of pediatric patients with CKD. Children and adolescents with CKD demonstrated significantly lower HRQoL, poorer oral health outcomes, and suboptimal oral hygiene practices compared to non-CKD controls. Based on these results, the null hypothesis of the study was rejected. To date, only a limited number of studies have simultaneously examined oral health parameters and HRQoL in children with CKD [2]. The only comparable study, conducted in Brazil by Silva et al. [2], utilized the PedsQL instrument and a restricted set of oral health indices. The present study extends this line of research by employing the KINDL questionnaire — a validated, age-specific instrument including both child self-reports and parent proxy-reports — alongside a comprehensive battery of oral health indices (ICDAS II, DDE, MIH, OHI-S, MGI) in Turkish children and adolescents with CKD, including patients undergoing dialysis and kidney transplantation. To our knowledge, this is the first study to investigate the association between oral health and HRQoL in children with CKD in Türkiye.

For caries assessment, the DMFT/dft indices used in the present study evaluated only cavitated lesions visible to the naked eye and thus provided limited information regarding lesion activity, progression, and treatment needs. To overcome these limitations, the ICDAS II index was additionally employed to enable a more detailed and reliable assessment of the severity and activity of carious lesions at various stages [31]. The prevalence and severity of dental caries were found to be lower in the CKD group compared to the non-CKD group. According to ICDAS II scores, severe carious lesions were more frequently observed in non-CKD children than in those with CKD. These findings are supported by recent studies reporting a lower caries prevalence in children and adolescents with CKD compared to their non-CKD counterparts [32, 33]. Despite high consumption of cariogenic foods and inadequate oral hygiene, the reduced caries burden observed in the CKD group is likely attributable to the protective effects of elevated salivary urea levels, which increase salivary pH and thus inhibit demineralization [32].

Amelogenesis, the process of enamel formation, is highly sensitive to systemic, genetic, and environmental influences. Disruptions in this process can result in DDE, which may present as demarcated or diffuse opacities, enamel hypoplasia, or MIH [34]. Enamel defects of systemic origin are particularly common in early stages of CKD and often affect multiple teeth simultaneously [12]. In line with previous research, the current study found a significantly higher prevalence of DDE in children with CKD compared to non-CKD controls [12, 13, 35, 36]. These mineralization defects—typically manifesting as demarcated or diffuse opacities and/or enamel hypoplasia—are thought to result from systemic disturbances in mineral metabolism commonly observed in CKD, such as hypocalcemia, decreased serum 1,25-dihydroxycholecalciferol, elevated serum phosphate, parathyroid hormone, and fluoride levels [12].

In the present study, children and adolescents with CKD exhibited a higher prevalence of plaque and calculus accumulation, along with an increased incidence of moderate to severe gingival inflammation. The relationship between CKD and gingival inflammation remains a topic of debate in the literature. Contrary to our findings, Serni et al. reported that immunosuppression and chronic uremia may not impair the gingival tissue’s defense against microbial dental plaque and could even suppress gingival responses, potentially resulting in reduced inflammation in individuals with CKD [37]. In addition, oral hygiene behaviors—including tooth brushing frequency and dental floss use—were significantly lower among participants with CKD. These results are consistent with previous studies indicating that chronic systemic conditions can negatively influence oral hygiene practices [12, 13, 38]. The reduced frequency of annual dental visits observed in children with CKD may be explained by the time constraints associated with the ongoing treatment burden. Moreover, additional barriers such as financial difficulties, lack of motivation, and psychosocial stress have also been identified as limiting access to dental care [39].

In recent years, with advancements in dialysis and renal transplantation, improving quality of life has become a central goal in the management of pediatric CKD [40]. However, validated and reliable tools for assessing overall HRQoL in children remain limited. The KINDL questionnaire employed in this study is widely recognized for its simplicity, clarity, and ease of administration. It includes age-appropriate versions for both children/adolescents and their parents [28]. According to the results obtained using this instrument, children and adolescents with CKD reported significantly lower HRQoL scores across all domains—including physical well-being, emotional well-being, self-esteem, family, friends, and school functioning—compared to non-CKD controls. These findings align with previous research by Ruidiaz and Higuita, who also reported reduced HRQoL scores in children with CKD, particularly in the physical, emotional, social, and school-related dimensions [41].

Interestingly, self-reported self-esteem scores among children with CKD were lower than the parent-reported scores, possibly reflecting a greater psychological burden perceived by the children themselves. Furthermore, disease perception scores were lower among patients compared to their parents, suggesting that families may maintain a more optimistic perspective regarding the impact of the disease [42]. In contrast, a recent meta-analysis reported that children with CKD tend to perceive an improvement in their quality of life over time—a trend not reflected in parental assessments [43].

Notably, HRQoL scores in the current study did not differ significantly according to CKD stage, dialysis, or transplantation status. Although renal transplantation is generally expected to improve quality of life, the absence of a significant difference is noteworthy. This finding may be explained by persistent challenges following transplantation, such as overprotective parental behavior, fear of graft rejection, and the ongoing need for frequent hospital visits in the post-transplantation period [17].

CKD affects various aspects of oral health in children and adolescents, including dental caries, developmental enamel defects, oral hygiene status, plaque and calculus accumulation, and the resulting gingival inflammation [13]. Furthermore, oral health—being an integral component of HRQoL—is of particular importance in this patient population, as it reflects overall health and well-being. In this context, Gomes et al. emphasized that among sociodemographic variables, higher socioeconomic status and better oral hygiene practices are key determinants of improved HRQoL, particularly in children and adolescents [44].

In the present study, multiple linear regression analysis identified CKD status, number of siblings (as a sociodemographic factor), and oral health indicators such as the OHI-S and the MGI as significant predictors of HRQoL in children and adolescents with CKD. The HRQoL of patients with CKD was found to be 24.4% lower compared to non-CKD individuals, highlighting the substantial impact of CKD on their daily lives. Interestingly, although higher OHI-S scores indicate poorer oral hygiene, an increase in total HRQoL scores was also observed. This seemingly contradictory finding may be attributed to the fact that children with more pronounced oral health problems are more likely to receive increased attention from caregivers and healthcare providers, potentially leading to a more favorable perception of their overall well-being. Alternatively, this result may suggest the influence of unmeasured variables—such as parental support or access to dental and medical services—that were not accounted for in the regression model.

In contrast, even very mild to mild levels of gingival inflammation were significantly associated with lower HRQoL scores, supporting the notion that early periodontal changes may have a measurable negative impact on children’s perceived quality of life. In line with these findings, Silva et al. reported that among patients with CKD, the physical and emotional subdomains of HRQoL were particularly affected by oral health conditions, especially in the presence of increased gingival inflammation and enamel defects [2]. The impact of oral health parameters on HRQoL may be largely attributable to the subjective nature of symptom perception related to both systemic and oral health conditions.

Overall, this study demonstrates that CKD imposes a considerable burden on oral health and that affected individuals tend to exhibit poorer oral health status and reduced HRQoL, particularly in relation to specific oral health indicators. These perceived symptoms may adversely affect physical, psychological, and social functioning [45]. Such outcomes may be partly explained by the tendency of children with CKD and their families to prioritize the management of the systemic disease while neglecting or underestimating the importance of oral health. Enhancing oral health in this population not only contributes to improved physical functioning, but also supports communication, esthetics, and self-esteem. Accordingly, a multidisciplinary approach—fostering collaboration between dentists and pediatric nephrologists—is essential for promoting oral health and implementing early preventive interventions that ultimately aim to enhance overall quality of life.

This study has several limitations that should be acknowledged. First, the reliance on self-reported questionnaires may introduce social desirability bias, particularly among children, potentially affecting the accuracy of the reported outcomes. Second, the study sample consisted of a relatively small and homogeneous group of CKD patients recruited from a single center, which may limit the generalizability of the findings. Third, parental sociodemographic variables, such as educational level, socioeconomic status, oral hygiene habits, and employment status, were not collected in the present study. Given the evidence that parental factors can influence children’s oral health behaviors and HRQoL, future studies should incorporate these variables to provide a more comprehensive understanding of the determinants of HRQoL in pediatric CKD patients. Future research should consider multicenter, prospective, and longitudinal study designs that include participants from diverse sociocultural backgrounds and incorporate objective clinical assessments to complement self-reported data.

## Conclusion

Children and adolescents with CKD demonstrated significantly lower HRQoL scores compared to their non-CKD peers, with CKD status, number of siblings, gingival health, and oral hygiene indices identified as significant predictors of HRQoL. Although dental caries prevalence was lower in the CKD group, overall oral health was adversely affected, particularly in terms of plaque and calculus accumulation, developmental enamel defects, and gingival inflammation — all of which were significantly associated with reduced HRQoL. Poorer oral hygiene behaviors, including less frequent tooth brushing, limited flossing, and irregular dental visits, further contributed to this burden. Notably, significant discrepancies between child self-reports and parent proxy-reports were observed only in the self-esteem and disease perception subscales, highlighting the psychological dimensions of HRQoL in this population. These findings highlight the need for a multidisciplinary, quality-of-life-oriented approach that integrates regular HRQoL monitoring with early preventive dental care to improve overall well-being in children and adolescents with CKD. Furthermore, the findings of this study are intended to inform the future development of actionable interventions and direct referral pathways between pediatric nephrology and pediatric dentistry clinics, with the goal of improving both oral health outcomes and HRQoL in this vulnerable population.

## Data Availability

The data that support the findings of this study are available from the corresponding author (N.K.D.) upon reasonable requests.
